# Hypoxia is regulating enzymatic wood decomposition and intracellular carbohydrate metabolism in filamentous white rot fungus

**DOI:** 10.1186/s13068-020-01677-0

**Published:** 2020-02-24

**Authors:** Hans Kristian Mattila, Mari Mäkinen, Taina Lundell

**Affiliations:** 1 Department of Microbiology, Faculty of Agriculture and Forestry, Viikki Campus, University of Helsinki, 00014 Helsinki, Finland; 2 Present Address: VTT Technical Research Centre of Finland Ltd, 02044 VTT Espoo, Finland

**Keywords:** *Phlebia radiata*, *Basidiomycota*, Hypoxia, Lignocellulose, Biodegradation, Fungal metabolism, Gene expression, Bioethanol, Transcription factor, Carbohydrate-active enzymes

## Abstract

**Background:**

Fungal decomposition of wood is considered as a strictly aerobic process. However, recent findings on wood-decaying fungi to produce ethanol from various lignocelluloses under oxygen-depleted conditions lead us to question this. We designed gene expression study of the white rot fungus *Phlebia radiata* (isolate FBCC0043) by adopting comparative transcriptomics and functional genomics on solid lignocellulose substrates under varying cultivation atmospheric conditions.

**Results:**

Switch to fermentative conditions was a major regulator for intracellular metabolism and extracellular enzymatic degradation of wood polysaccharides. Changes in the expression profiles of CAZy (carbohydrate-active enzyme) encoding genes upon oxygen depletion, lead into an alternative wood decomposition strategy. Surprisingly, we noticed higher cellulolytic activity under fermentative conditions in comparison to aerobic cultivation. In addition, our results manifest how oxygen depletion affects over 200 genes of fungal primary metabolism including several transcription factors. We present new functions for acetate generating phosphoketolase pathway and its potential regulator, Adr1 transcription factor, in carbon catabolism under oxygen depletion.

**Conclusions:**

Physiologically resilient wood-decomposing *Basidiomycota* species *P. radiata* is capable of thriving under respirative and fermentative conditions utilizing only untreated lignocellulose as carbon source. Hypoxia-response mechanism in the fungus is, however, divergent from the regulation described for *Ascomycota* fermenting yeasts or animal-pathogenic species of *Basidiomycota.*

## Background

Decomposition leading to utilization of plant biomass lignocelluloses by filamentous *Ascomycota* and *Basidiomycota* fungi is regarded as an aerobic process, since fungal respirative metabolism requires oxygen. Specifically oxygen is required to gain ATP for cellular metabolism and hyphal growth, as well as for active expression and secretion of an array of enzymes and metabolites necessary for the decomposition of plant cell wall biopolymers (cellulose, hemicelluloses, pectin, lignin) [1–3]. Furthermore, wood-decomposing fungi generate an oxidative first phase of early decay upon hyphal colonization of their solid lignocellulose habitat. This process generates reactive oxygen species (ROS) and produces specific extracellular redox enzymes against lignocellulose components [4–6]. In white rot fungi, enzymatic attack on wood lignin and crystalline cellulose microfibrils require specific redox enzymes dependent on molecular oxygen or ROS initiators like hydrogen peroxide [2, 4, 7].

*Basidiomycota* fungi are the main inhabitants and decomposers of dead wood and wood debris in the boreal and temperate forests ecosystems, and these fungi are responsible for generating either white or brown rot in wood [8, 9]. Brown rot decay may occur in construction wood [10] and in standing tree trunks in the forests, while white rot decay is more common in fallen trunks and wood subjected to moist soil conditions [2, 7, 9]. Wood-decaying saprobic fungi elongate their hyphae inside dead wood, and the fungi may confront rain, or flooding in fallen waterlogged trunks. White rot may be encountered even in saline coastal areas such as mangrove forests [11].

Therefore, we hypothesized that under the wet conditions of their natural habitats, the fungal hyphae may encounter situations of limited oxygen availability. It is thus likely that these organisms temporarily tolerate microaerophilic to anaerobic growth environments by switching to fermentative metabolism while they decompose wood. In fungi, production of ethanol by sugar fermentation is apparently a conserved trait that is not restricted to only the *Ascomycota* yeasts like *Saccharomyces cerevisiae* [12, 13].

As an example of this metabolic ability among wood-decaying fungi, white rot species of the taxonomic order *Polyporales*, genus *Phlebia* have shown great potential for ethanol fermentation from untreated lignocellulose [11, 14–16]. The phlebioid fungi are able to decompose both the wood carbohydrates and lignin moieties via secretion of a wide array of carbohydrate-active enzymes (CAZy [17] http://www.cazy.org/) and lignin-modifying oxidoreductases [4, 18]. However, apart from their ethanol fermentation ability little is known about the actual processes and regulation of the fermentative and primary metabolism of the white rot fungi.

Our previous proteomic and transcriptomic study of the phlebioid species *P. radiata* indicated time-dependent expression and the potential for co-regulation of several CAZy encoding genes [19]. In this study, we aimed to explore overall gene expression as well as regulation of the fungal metabolism under fermentative and ethanol-producing growth conditions.

We discovered that both enzymatic decomposition of wood lignocellulose and subsequent accumulation of extracellular ethanol occur under hypoxic conditions. These findings offer biological explanations for how variable environmental conditions affect the decomposition of wood lignocellulose and plant biomasses. Moreover, specific connections between genetic regulation of fungal extracellular and intracellular metabolism may be emphasized upon oxygen depletion. We also present a unique fungal metabolic pathway for adaptation to hypoxia.

## Results

### Extracellular decomposition of lignocellulose upon oxygen depletion

The role of lignocellulose and oxygen depletion was studied by analyzing in total 14 transcriptomes of *P. radiata*, derived from five different cultivation conditions. The RNA-Seq data were deposited to Gene Expression Omnibus [20] and is accessible through GEO accession number GSE141153 https://www.ncbi.nlm.nih.gov/geo/query/acc.cgi?acc=GSE141153. The 14 cultivations were named as Malt extract 1–3; Spruce 2 weeks 1 and 3; Spruce 4 Weeks 1–3 [4] followed by Spruce + board aerobic 1–3; and Spruce + board anaerobic_1–3 (this study). “Spruce 2 and 4 weeks” accompanied with “Spruce + board aerobic” represent lignocellulose containing cultivations with normal atmosphere. “Spruce + board anaerobic” is identical to “Spruce + board aerobic” except for the cultivation atmosphere. Fermentative conditions were created by sealing vials of “Spruce + board anaerobic” with rubber plugs. “Malt extract” is a liquid cultivation with no lignocellulose substrate.

Gas contents of the atmospheres of “Spruce + board aerobic” and “Spruce + board anaerobic” were measured over time during the cultivation. Cultivation flasks of “Spruce + board anaerobic” that were sealed with impermeable rubber plugs contained only 1.1 (± 0.2) % (v/v) of oxygen (O_2_) after 7 days of incubation. Oxygen content was found to be stabile similar in following time points day 14, 21 and 49. As expected the atmosphere of “Spruce + board aerobic” cultivation had approximately 21% (v/v) of oxygen throughout the cultivation (Additional file 1: Table S1).

**Fig. 1:**
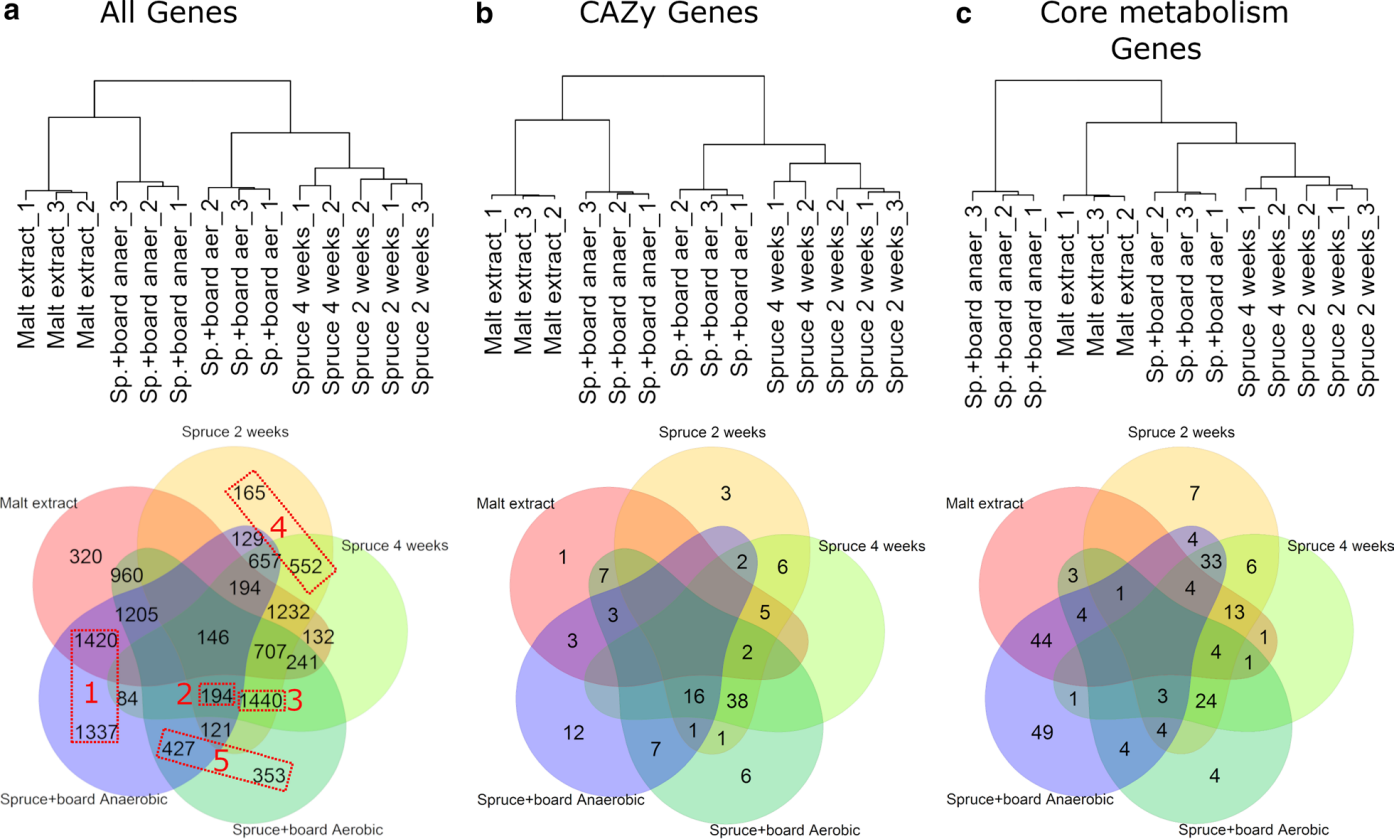
Clustering of *P. radiata* transcriptomes. Top: hierarchical clustering of all 14 transcriptomes. Sp., spruce; anaer, anaerobic; aer, aerobic. Below: Venn representation of upregulation patterns in relation to the growth substrate. **a** Clustering based on the expression of 12,017 differentially expressed genes. **b** All 113 CAZy genes. **c** All 216 Core metabolism genes. Biological explanation for the boxed Groups 1–5 (in red) in **a** is provided in the main text

Based on hierarchical clustering of VST normalized values of these transcriptomes, we found that expression of all the 12,017 differentially expressed genes formed two major groups (Fig. 1a). Fermentative conditions on lignocellulose (“Spruce + board anaerobic 1–3”, this study) and in the stationary liquid malt extract cultures (Malt extract 1–3) [4] formed two distantly related groups while aerobic conditions on lignocellulose substrates (“Spruce + board aerobic”, this study; Spruce 2 weeks and 4 weeks) [4] formed one main gene expression group. The CAZy encoding genes clustered according to cultivation atmosphere or growth substrate similar to all 12,017 differently expressed genes were analyzed (Fig. 1a, b). The 215 selected core metabolic genes clustered into a different pattern: ethanol-producing anaerobic conditions on lignocellulose substrate formed a separate main cluster (Fig. 1c). In all cases, individual transcriptomes of the biological replicates were the most similar to each other (Fig. 1, top panels).

Clustering analysis was resumed by dividing the 12,017 differentially expressed genes into 50 clusters by the Mfuzz analysis (Additional file 1: Table S2). The Mfuzz C (center) value [21] from each culture condition was examined for the 50 Mfuzz clusters. The C-values were used to indicate which genes were induced (higher expression of transcripts) or repressed (lower expression of transcripts) under a specific cultivation condition atmosphere or substrate in relation to other cultivation conditions and substrates (induced: *C* value > 0; repressed: *C* value < 0) (Additional file 1: Tables S2 and S3). Mfuzz cluster number 47 was removed from the analysis due to high variance and low coverage. As a result, five sections of sets of induced genes were obtained (Fig. 1a, lower panel).

We combined biological explanations with members of certain Venn diagram cluster and intersections (Fig. 1a, red rectangles). Genes induced (Mfuzz *C* > 0) during fermentation on ‘Spruce + board Anaerobic’ (1337 genes) combined with genes induced on ‘Malt extract’ (1420 genes) summed up to 2757 genes (Fig. 1a, lower panel). Together these sets of genes were described as Group 1, of ‘Anaerobically induced genes’. The second cluster of 194 genes included genes that were induced on all lignocellulose-containing substrates. Hence Group 2 was named ‘Lignocellulose-induced genes under both atmospheres’. Group 3 includes the 1440 upregulated genes shared with cultivations ‘Spruce + board Aerobic’, ‘Spruce 2 weeks’ and ‘Spruce 4 weeks’. This group was called ‘Lignocellulose-induced genes under aerobic conditions’. Group 4 ‘Spruce wood substrate induced genes’ includes 717 genes; and Group 5 ‘Spruce wood + core board substrate induced genes’ includes 780 genes induced on the mixture of the lignocellulose waste substrates under aerobic and fermentative atmospheres.

Group 1 is the largest by number containing most of the induced genes among the core metabolism genes (Fig. 1c). Up to 93 of the 216 studied genes belong to Group 1 (anaerobically induced genes). This group, however, has little representation of CAZy genes, as only 15 out of the 113 CAZy genes clustered into Group 1 (Fig. 1b). The simultaneous presence of oxygen and lignocellulose is seemingly important for promoting expression of both CAZy encoding genes as well as genes involved in and core metabolism, since Group 3 was well represented in both cases (38 and 24 induced genes, respectively). Group 2 had 16 induced CAZy genes together with three induced genes involved in core metabolism. These three genes (plus.g1220, minus.g2306 and plus.g1349) appear to encode non-secreted, intracellular β-glucosidases (Additional file 1: Table S4). In brief, these findings imply that atmospheric changes have a major impact on expression of fungal CAZy genes.

### Expression of CAZy genes under different atmospheric conditions

We analyzed the functional distribution of CAZy genes in each gene expression group. Surprisingly, of the 15 CAZy genes in Group 1, 12 genes were induced under the fermentative conditions on lignocellulose substrate, whereas malt extract medium induced expression of three genes. The 15 CAZy genes induced in Group 1 include 10 genes encoding cellulose-active enzymes (Table 1). Interestingly, three of these genes encode AA9 lytic polysaccharide monooxygenases (LPMOs), which are considered to require oxygen or hydrogen peroxide for catalytic activation [22]. Group 3 (lignocellulose-induced under aerobic conditions) included most of the expressed CAZy genes, comprising five AA9 LPMOs and many genes for activities against cellulose and hemicelluloses.

**Table 1:** Expression patterns of carbohydrate-active enzyme encoding genes of *P. radiata*

Cellulose active	CAZy	Hemicellulose active	CAZy	Pectin active	CAZy	Lignin active	CAZy
Group 1: anaerobically induced genes							
plus.g8293	AA9	plus.g13357	GH10	plus.g1493	GH28		
plus.g10695	AA9	minus.g4431	GH31	plus.g10556	GH105		
minus.g531	AA9	plus.g1199	GH31				
minus.g2565	GH5_5						
minus.g2003	GH7						
plus.g2026	GH7						
minus.g8589	GH7						
plus.g13166	GH9						
minus.g170	GH45						
plus.g7035	GH45						
Group 2: lignocellulose-induced genes under both atmospheres							
minus.g10273	AA9	plus.g12321	CE1	minus.g3209	GH28		
plus.g9320	AA9	minus.g10669	CE15				
minus.g8537	GH5_5	minus.g10025	CE16				
minus.g2832	GH5_22	minus.g319	GH5_7				
plus.g2874	GH5_22	plus.g3697	GH11				
plus.g4342	GH6	minus.g5676	GH35				
minus.g169	GH45	minus.g7290	GH74				
plus.g5796	GH131						
Group 3: lignocellulose-induced genes under aerobic conditions							
plus.g9628	AA3_1	minus.g2146	AA5	plus.g11923	CE8	plus.g1419	MnP1-long
minus.g10274	AA9	minus.g1785	CE15	plus.g5896	CE8	plus.g10562	MnP2-long
minus.g3552	AA9	minus.g3957	CE16	plus.g6259	GH28		
plus.g11539	AA9	plus.g637	CE16	minus.g11678	GH28		
plus.g11538	AA9	minus.g9090	CE16	minus.g10006	GH28		
plus.g13374	AA9	plus.g9565	GH2	minus.g4364	GH53		
minus.g2505	GH3	plus.g7205	GH5_7	minus.g8873	PL4		
plus.g7451	GH5_5	minus.g11036	GH10				
minus.g2568	GH5_5	minus.g12190	GH10				
plus.g8163	GH7	minus.g11656	GH10				
minus.g5721	GH12	minus.g11034	GH10				
plus.g1671	GH44	minus.g5675	GH35				
minus.g9081	GH131	minus.g4481	GH43				
		plus.g1633	GH51				
		minus.g9719	GH95				
		minus.g5364	GH115				
Group 4: spruce wood substrate induced genes							
minus.g5595	GH7	minus.g7380	CE1			minus.g3073	LiP1
		plus.g12991	GH2			minus.g6827	LiP2
		minus.g11037	GH10			plus.g11059	LiP3
						plus.g453	MnP3-short
						minus.g3827	MnP6-long
Group 5: spruce wood + core board mixture substrate induced genes							
plus.g9738	GH12	minus.g3390	GH5_7	plus.g1505	GH28		
		plus.g8530	GH27	minus.g11677	GH28		
		minus.g9779	GH29	plus.g9417	GH78		
		plus.g11941	GH35	minus.g2361	GH78		
		minus.g11462	GH43	plus.g9742	GH105		
		minus.g10886	GH74	minus.g12950	GH105		

Division of CAZy genes into five groups of differentially expressed genes is presented in Fig. 1. Genes are listed according to enzyme function and substrate specificity. CAZy genes were annotated previously [4, 19]

The majority of the pectin-active enzyme-encoding genes were included in Group 3 (Table 1). As expected, aerobic conditions induced the expression of lignin-active AA2 peroxidases (two genes for long-manganese peroxidases). Notably the AA2 peroxidases were absent from Group 2 (lignocellulose-induced genes under both atmospheres), which in contrast contains several genes encoding cellulose-active enzymes.

Lignocellulose substrates induced the expression of CAZy genes; two minor clusters of gene expression (Groups 4 and 5) were obtained from spruce wood (Group 4, on cultivation weeks 2 and 4) and the mixture of spruce wood sawdust and core board (Group 5). Group 4 contains five of the *P. radiata* AA2 lignin-active enzyme genes including lignin and manganese peroxidases (LiPs, MnPs), together with three genes for hemicellulose activity and one GH7 cellobiohydrolase encoding gene (Table 1). In contrast, the lignocellulose mixture (spruce sawdust + core board) induced expression of six genes for pectin, six genes for hemicellulose degradative activity, but only one gene for cellulose degradative activity. Groups 1, 2 and 3 hold all of the endoglucanase-encoding CAZy GH5 genes (Table 1). The only laccase gene that has been detected also from proteome (plus.g7011) [4] was found to be repressed in fermentative conditions (Additional file 1: Table S4). In total, the five distinct gene expression groups explained expression of 81% (91 genes) of the 113 previously identified and functionally annotated *P. radiata* CAZy genes [19].

### Enzyme activity measurements

**Fig. 2:**
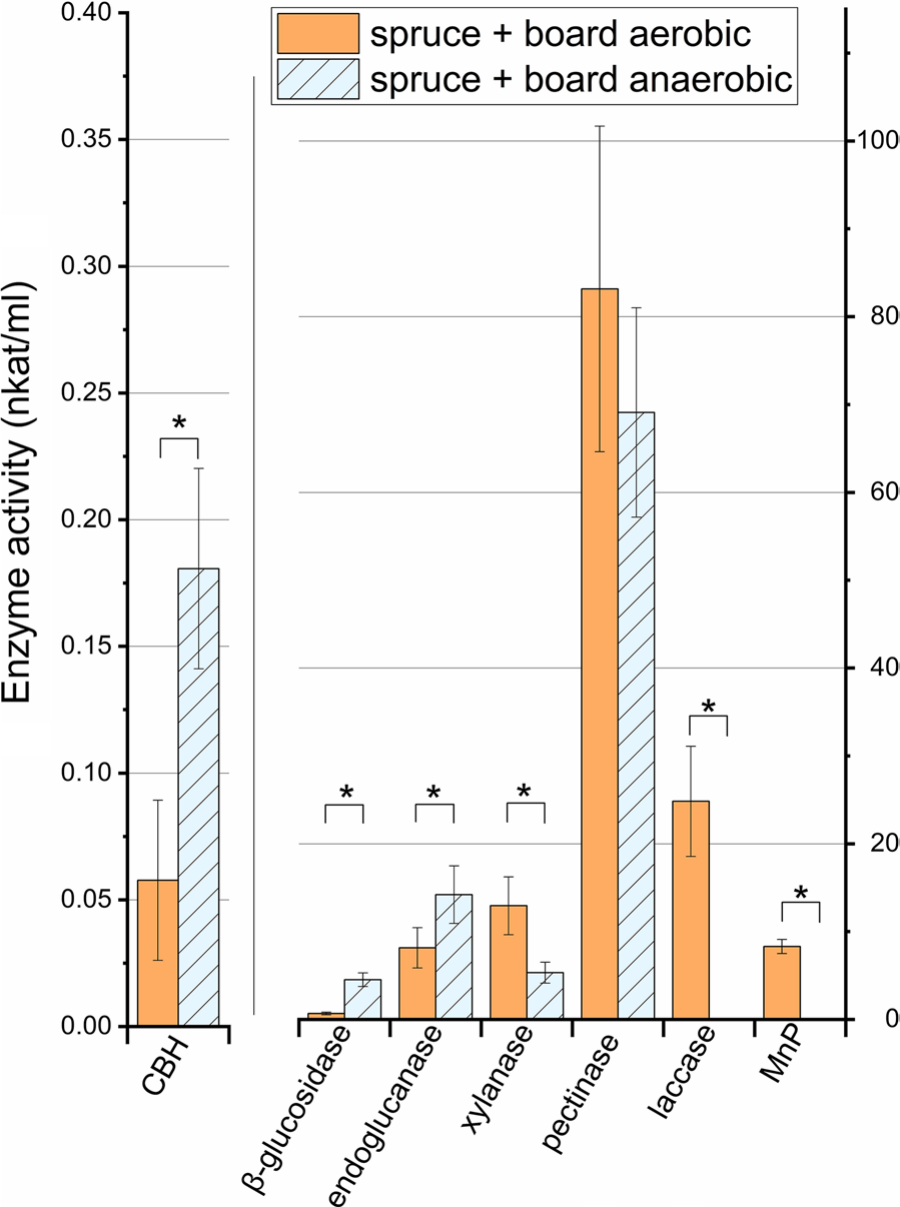
Enzyme activities of *P. radiata*. Comparison of cellulose, hemicellulose, pectin and lignin-active extracellular enzyme activities measured from cultivation day 14 on spruce + board aerobic and anaerobic. Notice the different *y*-axis for CBH at the left. Brackets with asterisk define statistical differences between culture atmospheres (*p *< 0.01). CBH, cellobiohydrolase; MnP, manganese peroxidase. Bars represent the mean value of three parallel culture flasks while error bars represent standard deviation

Activities of extracellular β-glucosidase, endoglucanase, xylanase, pectinase (polygalacturonic acid depolymerization assay), manganese peroxidase and laccase enzymes were measured from the fungal cultures on spruce + core board substrate to confirm the findings of gene expression. Activities of cellulose-active enzymes (endoglucanase, β-glucosidase and cellobiohydrolase CBH) were higher under fermentative conditions (spruce + board anaerobic) while the activities of xylanase and lignin-modifying enzymes were the opposite (Fig. 2). Depolymerization activity of polygalacturonic acid showed no difference between the cultivations. Enzyme activities normalized with total RNA showed that all other activities were significantly higher at fermentative conditions except laccase and MnP activities that were significantly higher in aerobic conditions (for total RNA-quantities, see “Materials and methods” RNA-Seq and transcriptome assembling). Surprisingly, β-glucosidase enzyme activity increased under fermentative conditions, although there was only one upregulated β-glucosidase encoding gene (minus.g7505) upon fermentative cultivation conditions (Additional file 1: Table S3). The enzyme encoded by this gene is also the only β-glucosidase that contains a CBM module.

### Promoter region analysis of the CAZy genes

To find explanations for the expression patterns of the multiple and functionally divergent lignocellulose-decomposing CAZy encoding genes, we examined expression of known fungal DNA binding transcription factors such as ACE1-3, McmA, GaaR, XlnR, and Sxlr, which are regulators for genes coding for cellulose, hemicellulose and pectin degradative enzymes [23–26]. We looked for similar expression of the transcription factor-encoding genes and those CAZy genes predicted to possess the specific regulator binding sequence motifs on their promoter regions. This approach aimed to anticipate regulative factors that are effective for fungal extracellular and intracellular metabolism upon decomposition of lignocellulose.

In brief, we found no protein homologs of *P. radiata* for the ACE1-3, XlnR or Sxlr transcription factors described in plant biomass decomposing *Ascomycota*. Only a homolog for McmA activator was identified (*P. radiata* gene plus.g9031), which was noted to be induced during fermentation on the waste lignocellulose mixture but also on spruce wood after 2 and 4 weeks of cultivation. Although we found no homologs for ACE1-3, the binding motif 5′-GGCTAATAA-3′ for ACE2 [27] was present 655–647 bp and 651–643 bp upstream of the promoter regions of two oxygen-depletion-induced GH7 cellobiohydrolase encoding genes (minus.g2003 and plus.g2026, respectively) (Table 1). Interestingly, this specific motif was not detected on the promoter regions of any other *P. radiata* CAZy genes. Search for the *Aspergillus niger* pectin usage transcription factor GaaR [28] gave only two distant hits (genes minus.g11147 and minus.g9788). However, we found no GaaR binding motif on the promoters of *P. radiata* genes encoding pectin-active enzymes or genes involved in metabolism of galacturonic acid.

MEME search discovered an enriched motif 5′-SGTATAAA-3′ of unknown function from promoter regions of *P. radiata* genes. This motif was recognized on the promoter regions of four CAZy pectin degradation-encoding genes as well as three genes encoding specific intracellular NADPH-dependent reductases (enzyme functions [EC 1.1.1.372], [EC 1.1.1.365] and [EC 1.1.1.21]), most likely involved in the catabolism of carbohydrates released from pectin and hemicelluloses. In addition, promoter regions of all lignin-modifying AA1 and AA2 CAZy class genes possessed the motif 5′-SGTATAAA-3′. This motif located once 74–117 nucleotides upstream from the translational coding sequence start codon. A similar motif has been reported to be present on the promoters of lignin-active AA2 CAZy genes in white rot fungi [29].

### Carbohydrate consumption of *P. radiata* under varying atmospheres

**Fig. 3:**
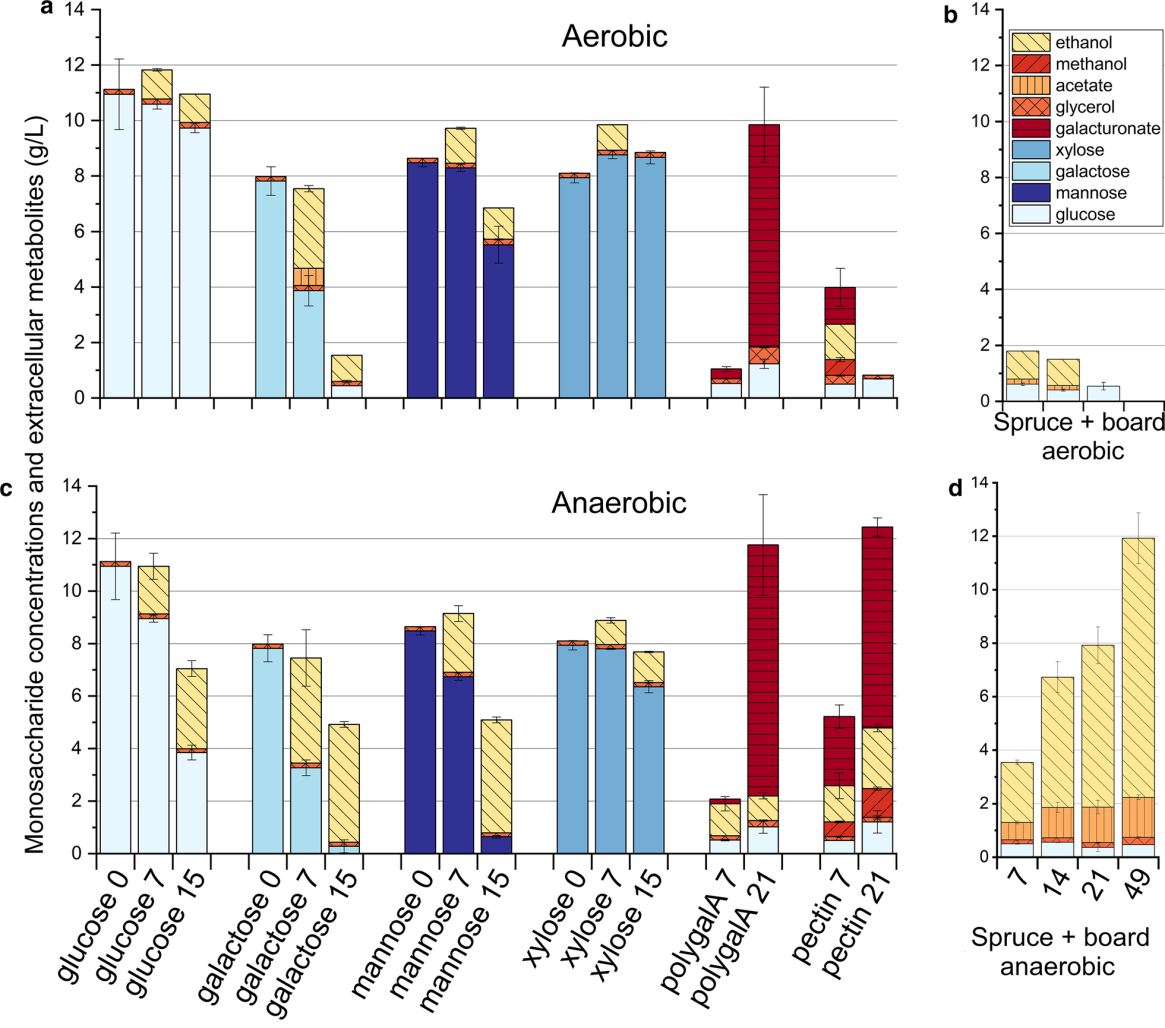
Cumulative display of consumption of carbohydrates and bioconversion of solid lignocellulose substrate by *P. radiata*. **a**, **c** Consumption of carbohydrates and accumulation of extracellular metabolites in stationary liquid cultures under aerobic (**a**) and anaerobic conditions (**c**). **b**, **d** Extracellular metabolites produced on the solid lignocellulose under aerobic “spruce + board aerobic” (**b**) and fermentative “spruce + board anaerobic” (**d**) conditions at different time points. Numbers on *x*-axis represent cultivation days. Bars represent the mean value of three parallel culture flasks while error bars represent standard deviation

To reveal how the intracellular carbohydrate-utilizing metabolism responded to the changes in culture atmosphere, we cultivated *P. radiata* on various monosaccharides (glucose, galactose, mannose, and xylose), and polysaccharides (polygalacturonic acid and pectin) as the sole carbon sources. Fungal mycelium used all studied monosaccharides as well as pectin and polygalacturonic acid as substrates under both cultivation atmospheres (Fig. 3a, c). Galactose was consumed fastest of the monosaccharides followed by mannose, glucose and xylose. From pectin, accumulation of galacturonic acid as degradation product was increased under fermentative conditions (Fig. 3c). Surprisingly, ethanol was also detected under aerobic conditions in cultivations on almost all of the studied carbohydrate substrates as well as from aerobic cultures on the solid lignocellulose “spruce + board aerobic” (Fig. 3a, b).

The intake and conversion of glucose and mannose was faster under fermentative atmosphere than under aerobic conditions (Fig. 3a, c). Galactose or xylose uptake showed no difference within 15 days between atmospheres. *P. radiata* converted the waste lignocellulose mixture (spruce wood sawdust + core board), leading to an accumulation of ethanol, up to 10 g/L in 49 days under fermentative conditions (Fig. 3d). In addition, we detected production of extracellular glycerol (0.14–0.27 g/L) and a moderate amount of acetate (0.6–1.5 g/L) from the lignocellulose substrate under fermentative conditions. We calculated the carbohydrate content of the substrate (1 g of core board [30] and 4 g of spruce wood sawdust [31] to estimate the quantity of utilizable hexose sugars in the substrates. Based on stoichiometry, we calculated the quantity of hexose sugars required to produce the detected quantities of ethanol glycerol and acetate. After 14 days of cultivating *P. radiata* under fermentative conditions on the lignocellulose substrate, 8.7% of its total hexose sugars was required to produce 4.8 g/L of ethanol, 1.13 g/L of acetate and 0.17 g/L of glycerol. Prolonging the cultivation up to 49 days resulted in 16.3% of hexose sugars to be converted into end products. Cultivation on natural-type pectin substrate under both aerobic and fermentative conditions led into slight accumulation of methanol (Fig. 3a, c).

### Intracellular metabolism under hypoxia

**Fig. 4:**
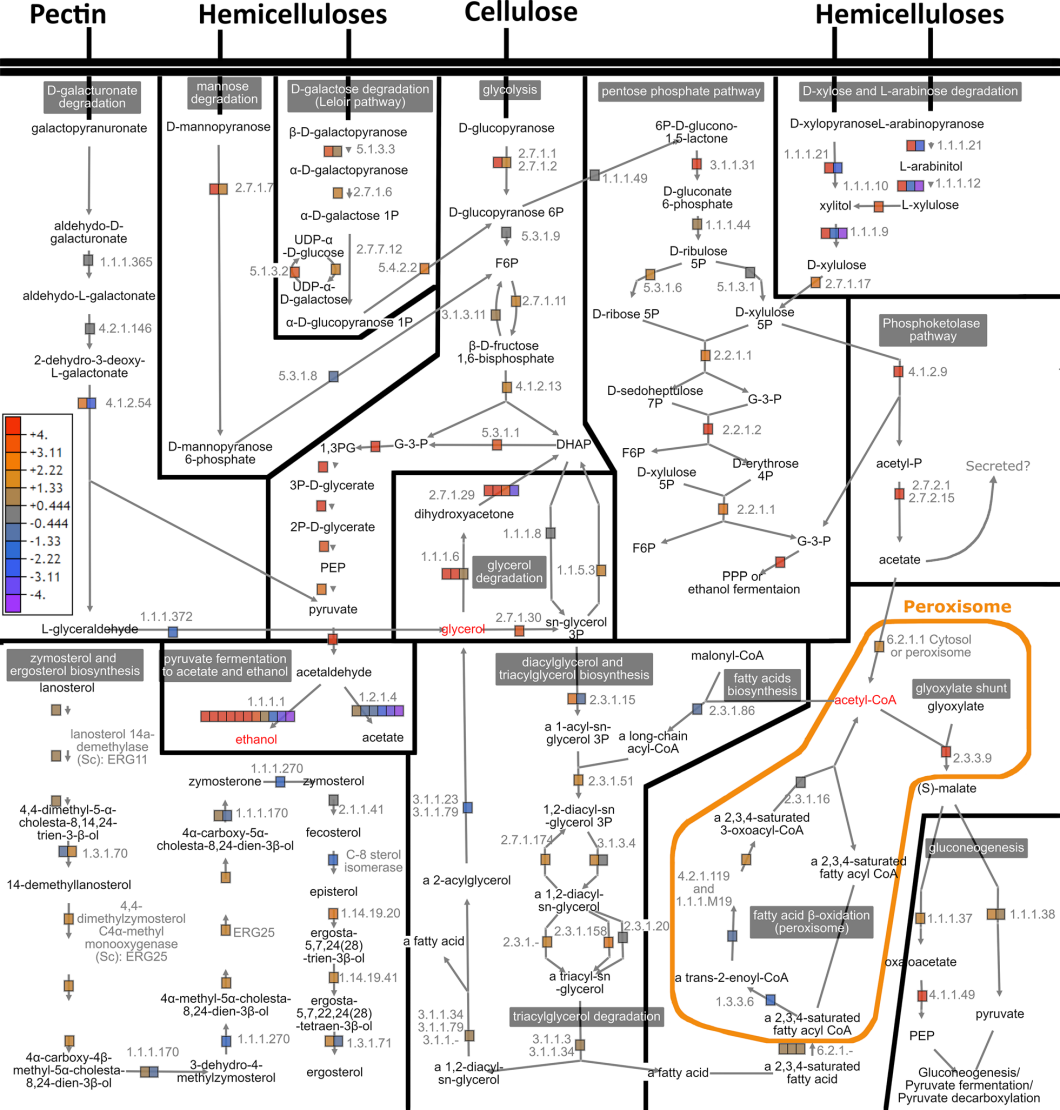
Expression of intracellular metabolic pathways in *P. radiata*. Pathways represent the catabolism of lignocellulose-derived carbohydrates combined with biosynthesis of ergosterol, and lipid and triglyceride metabolism. Each colored square represents level of transcriptional expression of an individual gene under fermentative conditions. Gray to orange squares = genes upregulated in hypoxia. Gray to purple squares = genes downregulated in hypoxia

Oxygen depletion while growing on lignocellulose substrates affected the intracellular metabolism of *P. radiata* by inducing expression of 93 core metabolic genes (Fig. 1c). Thus, we studied the impact of oxygen depletion leading to hypoxia on expression of genes of intracellular metabolism by calculating the transcriptome fold changes between conditions of ‘Spruce + board Anaerobic’ and ‘Spruce + board aerobic’. Genes encoding proteins involved in the intracellular primary metabolism of glycolysis and Leloir pathways as well as glycerol metabolism and triglyceride degradation and biosynthesis and phosphoketolase pathway, were upregulated under hypoxia (Fig. 4) (Additional file 1: Table S4). Phosphoketolase pathway produces acetate/secreted acetic acid through acetyl phosphate (Fig. 4). In addition, genes involved in oxidative phosphorylation and especially those encoding mitochondrial complexes III, IV and ATP synthase were upregulated (Additional file 1: Table S4). In contrast, fatty acid β-oxidation, pectin degradation catabolism, and parts of ergosterol biosynthesis pathways were either constant in expression or repressed under hypoxia. Based on expression patterns of genes coding for ethanol dehydrogenases [EC 1.1.1.1] and aldehyde dehydrogenases [EC 1.2.1.4], pyruvate was most likely converted into ethanol instead of acetate (Fig. 4).

### *P. radiata* phosphoketolase pathway and acetyl kinase enzymes

We investigated more closely the phosphoketolase [EC 4.1.2.9] candidate protein encoded by the homologous *P. radiata* gene plus.g11264, which has been detected also as a peptide in a previous proteome study [4]. The translated protein candidate showed 36% amino acid sequence identity with the phosphoketolase of *Bifidobacterium breve* (D6PAH1, Uniprot accession) and 43% identity with *Cryptococcus neoformans* predicted protein (J9VR37, Uniprot) [32], respectively (Additional file 2). Protein folding structural similarity between *P. radiata* and *C. neoformans* models was 37%. Analysis of the translated *P. radiata* protein model plus.g11264 and J9VR37 adopting PHYRE2 Protein fold recognition server (http://www.sbg.bio.ic.ac.uk/~phyre2) revealed that the conserved binding sites for coordinated Ca-ion and the thiamine pyrophosphate (TPP) ligands were found in both *P. radiata* and *C. neoformans* enzyme models. Superposition of the 3D protein models turned out to be identical with the *B. breve* phosphoketolase crystal structure model 3AHC (RCSB protein database) (Additional files 3 and 4).

The second enzyme of phosphoketolase pathway, acetate kinase [EC 2.7.2.1] is responsible for the conversion of acetyl phosphate (product of phosphoketolase activity) to acetate. The translated predicted protein for acetate kinase gene (plus.g11263) demonstrated amino acid sequence identity of 37% with *C. neoformans* acetate kinase model (J9W3A6, Uniprot) that has been structurally characterized (crystal structure 4H0P, RCSB PDB database) [33]. Both the *P. radiata* protein model and *C. neoformans* acetate kinase enzymes possess the conserved ligand binding sites for ATP. Similar to phosphoketolase, these two proteins share an identical 3D structure based on homology modeling and superposition analysis.

### Transcription factors and regulation of primary metabolism under hypoxia

In order to understand the regulation behind carbohydrate metabolism, we focused on transcription factors involved in fungal metabolic pathways of carbon usage (Table 2) and ergosterol biosynthesis. We searched potential candidates for known transcription factors in *P. radiata* based on protein homology. We analyzed expression of transcription factor genes candidates by comparing their expression fold changes between aerobic and fermentative conditions, similarly as described above.

**Table 2:** Transcription factors of fungi involved in carbon metabolism

Function	Uniprot entry	Reference organism for protein homolog	Protein name	*P. radiata* ortholog	Effect of oxygen depletion
Ethanol regulated TF. activates PEP synthesis [34]	CNAG_04588	*C. neoformans*	ERT1	plus.g634	1.01
Non-fermentable carbon usage [35]	CNAG_02215	*C. neoformans*	Hap3	minus.g3547	− 1.54
Non-fermentable carbon usage [35]	CNAG_07680	*C. neoformans*	Hap5	minus.g13227	− 0.97
Carbon catabolite repression regulator [36]	J9W323 P27705	*C. neoformans* *S. cerevisiae*	CreA Mig1	plus.g8859	− 0.01
Gluconeogenesis activator [37]	P39113	*S. cerevisiae*	Cat8	plus.g8696	− 0.84
Alternative carbon usage [38]	P07248	*S. cerevisiae*	Adr1	minus.g541	3.2

Gene expressional change in hypoxia, fold change ≥ 1 upregulated under hypoxia

PEP, phosphoenolpyruvate; TF, transcription factor

The fungal transcription factor-encoding genes associated with non-fermentative carbon utilization were constitutively expressed between aerobic and fermentative conditions (fold change ≤ 1 and ≥ − 1) or down-regulated during fermentation (fold change ≤ − 1). As exceptions, the orthologous genes encoding transcription factor ERT1-like homolog of *C. neoformans* and Adr1-like homolog, both genes were upregulated under fermentative conditions. The predicted *P. radiata* gene for Adr1 shows remarkable induction under hypoxia (Table 2).

Ergosterol synthesis is crucial for sensing hypoxia in fungi, and has been reported to respond to low oxygen tension and other stress factors in the *Ascomycota* fission yeast *Schizosaccharomyces pombe* [39]. The closest ortholog of the fungal ergosterol biosynthesis regulator-element binding protein (SREBP) encoding gene in *P. radiata* (gene minus.g3490) was constitutive in expression under fermentative conditions. However, the identity of translated amino acid sequences is only 16% between *P. radiata* and the other *Basidiomycota* species *C. neoformans* Sre candidates.

**Fig. 5:**
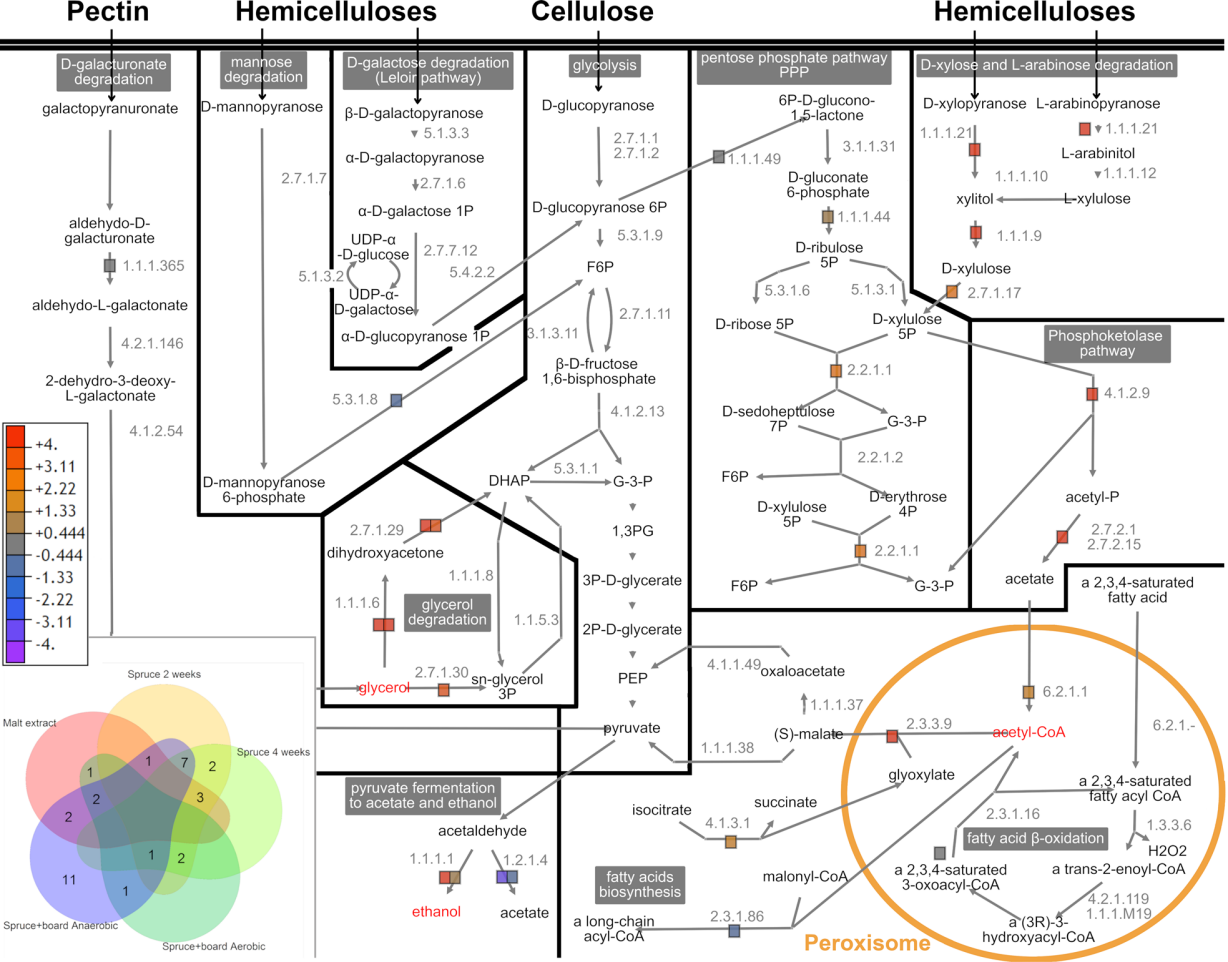
Distribution and expression of genes with promoter-regional Adr1 binding motif involved in carbohydrate metabolism. Orange to red squares = genes with Adr1 binding motif induced in hypoxia. Purple boxes = genes with Adr1 binding motif repressed in hypoxia. Gray boxes = genes constantly expressed under both atmospheres. Bottom left corner, Venn diagram: cultivation condition and substrate influenced induction of genes with promoter region Adr1 binding site motifs. Orange circle: peroxisomal reactions

Next, we analyzed the presence of the Adr1 transcription factor DNA binding motif 5′-TGCGGGGA-3′ [32] in promoter regions of *P. radiata* core metabolism and CAZy (Additional file 1: Table S4). Of the 33 genes involved in intracellular metabolism of *P. radiata* that demonstrated to possess the Adr1 transcription factor binding motif in 5′ → 3′ direction, up to 25 were induced during fermentation on the mixed lignocellulose substrate (‘Spruce + core board Anaerobic’) (Fig. 5). In addition, three of the CAZy GH7 genes contained the Adr1 binding motif on their promoter regions (Additional file 1: Table S4). Interestingly, promoters for genes active in xylose reductive pathway, pentose phosphate pathway (PPP), and phosphoketolase pathway, glyoxylate shunt and glycerol metabolism possessed this binding motif (Fig. 5).

## Discussion

Decomposition of wood and plant biomass lignocelluloses by *Basidiomycota* wood-decaying fungi is an extracellular process, requiring secreted hydrolytic and oxidoreductase enzymes (CAZymes and auxiliary enzymes) [2, 4, 6, 7, 40]. Therefore, these biological systems have been studied under aerobic experimental conditions. However, the ability to ferment carbohydrates and lignocelluloses into ethanol, particularly by the species of the genus *Phlebia* (order *Polyporales*) [15, 41] has pinpointed the need to explore the fungal metabolic pathways and their regulation under oxygen-depleted conditions. Our study is the first complete transcriptomic investigation that compares fermentative and aerobic cultivation conditions, in order to explain the cellular events of fungal decay of wood and lignocellulose concomitant with ethanol fermentation under hypoxia. *P. radiata* possess a wide array of secreted lignocellulose-decomposing enzymes [4, 18, 19], but in addition, it possesses the pathways required for the catabolism of the released galacturonic acid, pentose and hexose sugars (Fig. 4).

In other species of *Polyporales* white rot fungi such as *Phanerochaete chrysosporium*, *Phanerochaete carnosa*, *Cerrena unicolor*, and *Pycnoporus cinnabarinus*, the type of wood and lignocellulose substrate directs the transcriptional expression of CAZy genes [42–45]. In our study on *P. radiata*, we found that atmospheric transition is the key regulator of CAZy genes encoding cellulose-active enzymes. The composition of the carbon source or substrate regulated only two cellulose-active CAZy genes (Groups 4 and 5). In contrast, expression of up to 23 *P. radiata* CAZy genes encoding cellulose-active enzymes was regulated by transition of the culture atmosphere from aerobic to oxygen-depleted fermentative state (Groups 1 and 3).

Remarkably, hypoxic conditions induced the expression of up to 10 genes for cellulose-degradative CAZymes in *P. radiata*, which was also detected in enzyme activity measurements. It remains undefined whether LPMO activity affected the activity results as the enzyme activity measurement was conducted in aerobic conditions. Regarding decomposition of plant cell wall lignocelluloses, CAZy enzyme families GH6 and GH7 of cellobiohydrolases are important for cleaving crystalline regions of cellulose chains [2, 7]. Of the six GH7 genes of *P. radiata* [4, 19], hypoxia induced expression of three genes (in Group 1). The other GH7 genes and the single GH6 gene clustered to the other gene expression groups. Since CBH enzyme activity was detected under both atmospheric conditions, therefore it is thereby plausible that all six predicted GH7 enzymes of *P. radiata* have an important role in lignocellulose degradation.

Regarding lytic polysaccharide mono-oxygenases, out of the 12 genes encoding *P. radiata* AA9 LPMOs [19], seven genes were induced by lignocellulose (five in Group 3, and two in Group 2). To our surprise, fermentative conditions specifically induced three AA9 genes clustered to Group 1. Concerning the regulation of cellulose actin enzymes encoding genes, a homolog for McmA previously described in filamentous fungi [46] was the only candidate found in the *P. radiata* genome. Therefore, it is likely that regulation of the cellulose-active genes in this fungus is very different from the mechanisms reported for *Ascomycota* species, and requires further investigation.

Oxygen depletion repressed the expression of a number of hemicellulose-active CAZymes in *P. radiata*, which was consistent with detected extracellular xylanase enzyme activity. On the contrary, the growth substrate apparently regulated the expression of pectin-degradation active CAZy genes. The mixture of spruce wood sawdust and core board induced the expression of genes encoding enzymes for rhamnose degradation. One of the GH28 enzyme-encoding genes (plus.g1493) was among the highest upregulated genes on comparing anaerobic to aerobic culture conditions (Spruce + board anaerobic and Spruce + board anaerobic), indicating an important role in pectin utilization by the fungus under fermentative conditions. This is concurrent with the finding that the fungus demonstrated polygalacturonic acid depolymerizing activity and consumption under both atmospheric conditions, and was able to utilize this carbohydrate polymer as a carbon source under fermentative conditions. Galacturonic acid seemed to accumulate into the cultivation liquid as degradation product from pectin and polygalacturonic acid. Lack of detection of the product from pectin under aerobic atmosphere may be due to inefficient degradation of pectin or efficient intake and consumption of galacturonic acid. *P. radiata* is a wood-decomposing white rot fungus [4] that encounters pectin polysaccharides in its natural substrate (dead wood), mainly in the middle lamellae of xylem wood cells. Noticeably, various GH28 pectin-active CAZymes have been detected in the proteome of the fungus expressed on spruce wood [4]. Culture liquids under fermentative conditions possessed no laccase or MnP activity, which was consistent with the transcriptome analyses demonstrating the lack of stimulation for expression.

Our results point towards co-regulation of the fungal intracellular primary metabolism and lignocellulose-decomposing, secreted CAZymes. Extracellular pectin-degradation active genes and genes responsible for intracellular catabolism of galacturonic acid were both assigned to Group 3. This indicates that the extracellular and intracellular metabolic reactions and pathways for pectin degradation may operate under shared regulation as is reported for species of *Aspergillus* [47]. However, despite the noticed co-expression of enzyme-encoding genes involved in pectin decomposition and conversion of galacturonic acid, we could not detect *Aspergillus* GaaR transcription factor binding site [47] within *P. radiata* promoter regions. In fact, we found no such enriched motifs on the promoters of pectin metabolic genes, which could have explained this co-expression. Understanding the shared regulation between lignocellulose-degradative CAZyme encoding genes and intracellular metabolic pathways is necessary for microbial biotechnology approaches aiming at fungal applications. Efficient bioconversion of the secondary carbohydrate polysaccharides of lignocellulose, that is hemicelluloses and pectins, is mandatory for establishing sustainable second-generation production of bioethanol.

### Adaptation to hypoxia by regulation of intracellular metabolism

Based on the large number of upregulated genes shared between “Spruce + board anaerobic” and “Malt extract” cultivations, we concluded that the submerged mycelium might encounter hypoxia under non-agitated conditions. Similar outcome was recently reported for the *Ascomycota* species *Cordyceps militaris* [48]. This is consistent with minor ethanol accumulation detected from liquid media under aerobic cultivations. Although, ethanol was also detected under aerobic cultivations we determined that *P. radiata* is a “Crabtree-negative” organism based on the induction of glycolysis under hypoxia or alternatively, repression of metabolic genes functional in glycolysis and fermentation under aerobic conditions. In the budding yeast *S. cerevisiae*, a “Crabtree-positive” effect refers to the ability to produce ethanol under aerobic conditions and high glucose concentrations which is seen also in gene expression [49, 50]. These results reinforce the primary interpretation of our results that upon wood decomposition, wood-decaying fungi encounter partial hypoxia, leading to fermentation and ethanol production.

Hypoxia does not repress the catabolism of carbohydrates in *P. radiata*. Transcription factor CreA and catabolic pathways involved in less favorable carbon sources of *P. radiata* were not induced under hypoxia. Therefore, we concluded that concentration of glucose or other carbohydrates was not high enough to induce carbon catabolite repression [51] under hypoxia. Expression of the diauxic shift-associated transcription factor Cat8 candidate in *P. radiata* [37] followed a similar pattern to CreA. In *S. cerevisiae*, Cat8p targets a gluconeogenesis-related fructose-bisphosphatase [EC 3.1.3.11], which was constitutively expressed in *P. radiata*. In contrast to CreA and Cat8 homologs, the expression of transcription factor ERT1 and its target gene phosphoenolpyruvate carboxykinase [EC 4.1.1.49], were induced in *P. radiata* under fermentative conditions. In conclusion, we suggest that *P. radiata* is enforcing catabolism of hexose and pentose sugars in hypoxia state. Based on differential gene expression, less favorable carbon sources such as glycerol, TCA-derived organic acids, and acetate are simultaneously transformed into PEP and pyruvate that are likely routed to ethanol fermentation instead of gluconeogenesis (Fig. 4).

Stimulation of ergosterol synthesis is a known adaptation to hypoxia in fungi such as the *Ascomycota* fission yeast *S. pombe*, and *Basidiomycota* human-opportunistic pathogen *C. neoformans* [39, 52]. As our data show, expression of ergosterol biosynthetic pathway genes was, partially induced in *P. radiata*. Interestingly all enzymes in this pathway that require molecular oxygen as cofactor are slightly upregulated fold change > 1. Several Basidiomycota species do not have Sre homolog [53]. Due to low homology between Sre candidates, it remains possible that *P. radiata* belongs to this group of fungi with different type of ergosterol regulation.

Glycerol metabolism of *P. radiata* was highly upregulated under hypoxia. This is noteworthy because, the culture media were not supplemented with glycerol, and extracellular glycerol production levels remained low throughout the experiments. Glycerol formation acts also as a parallel pathway to ethanol fermentation ensuring an adequate concentration of cytosolic NAD+, which ensures the functionality of glycolysis during hypoxia. Based on the expression data, glycerol was further utilized in triglyceride synthesis or mitochondrial glycerol phosphate shuttle [54]. We noticed that in addition to the activity of flavoprotein dehydrogenase [EC 1.1.5.3], which is required for the electron shuttle, oxidative phosphorylation complexes III, IV and V (ATP synthase) are induced under hypoxia. This shuttle transfers protons and electrons from DHAP into mitochondrial FAD thus regenerating even more NAD+ in cytosol. In conclusion, our findings concerning the expression of oxidative phosphorylation, glycerol, fatty acid and triglyceride metabolisms combined with ergosterol biosynthesis, which is not required for mitochondria biosynthesis [55], all point out that mitochondrial metabolism and biogenesis may have a central role under hypoxia.

### Phosphoketolase pathway and transcription factor Adr1 in *P. radiata*

It has been proposed that Adr1 regulates the use of non-fermentable carbon sources in fungi, by inducing genes involved in conversion of ethanol into acetyl-CoA and incorporating glycerol into glycolysis or gluconeogenesis [38, 56]. In addition, Adr1 has been associated with induction of aldehyde dehydrogenases, β-oxidation and induction of genes encoding catalases that play crucial roles for instance in β-oxidation by breaking down hydrogen peroxide [38, 56, 57]. Our results (Fig. 5) notably deviate from the previous studies on *Ascomycota* Adr1 using cultivation conditions of normal atmospheric oxygen levels, or on laboratory culture media containing for instance glycerol as carbon source.

Interestingly, we detected accumulation of secreted acetate in cultures on lignocellulose substrate under fermentative conditions where the NAD+/NADH ratio is usually low [58]. Since acetate production from ethanol or acetaldehyde via aldehyde dehydrogenase [EC 1.2.1.4] requires NAD+ we concluded, there might be alternative acetate producing routes. Homologs for other acetate producing enzymes (succinyl-CoA: acetate CoA-transferase [EC 2.8.3.18] and acetyl-CoA hydrolase [EC 3.1.2.1]) were not found in *P radiata*. Therefore, we suggest that phosphoketolase pathway is involved in the production of extracellular acetate as previously suggested in *C. neoformans* [32].

Promoters of *P. radiata* genes involved in the PPP and d-xylulose-5-phosphate phosphoketolase pathways carried the Adr1 binding motif. The motif is also present on promoters of genes involved in acetyl-CoA conversion leading to malate in the cytosol. Therefore, it remains unclear whether the activation of phosphoketolase pathway under hypoxia leads solely to production of acetate and ATP [32], or whether acetate and ATP are further transformed into acetyl-CoA [59]. In *P. radiata*, expression of mitochondrial acetyl-CoA synthetase [EC 6.2.1.1] encoding gene (minus.g1298) was constitutive while another gene (plus.g1345) encoding acetyl-CoA synthetase without any mitochondrial target peptide was upregulated. This indicates that during hypoxia *P. radiata* may convert part of the acetate into acetyl-CoA outside mitochondria, possibly in peroxisomes. Acetyl-CoA is required in peroxisomal glyoxylate shunt to generate malate, which can be transformed for instance to pyruvate. Based on differential gene expression in *P. radiata,* the source of peroxisomal acetyl-CoA is more likely the phosphoketolase pathway rather than β-oxidation. Therefore, we propose that phosphoketolase pathway may facilitate production of acetyl-CoA. Only few reports exist discussing the role of phosphoketolase pathway in eukaryotic organisms. However, hypoxia-associated upregulation of phosphoketolase pathway encoding genes was observed in human pathogen *C. neoformans* [60], which is in accordance with our results on *P. radiata*.

Interestingly, extracellular acetic acid was detected only on the cultures “Spruce + board anaerobic”, but not on liquid medium with monosaccharides as the sole carbon source. It has been suggested that production of acetate aids in decreasing extracellular pH in infections caused by *Cryptococcus* [32]. In the *Polyporales* wood-decaying fungi, increasing acidity is promoting lignocellulose degradation [2, 3, 5]. However, acidification of the environment is mainly due to production and secretion of oxalic acid [15, 61]. Under hypoxia, *P. radiata* requires phosphoketolase pathway for the biosynthesis of both of these acids.

In addition, the phosphoketolase pathway provides an alternative for PPP. Although the ATP yield per glucose is lower in comparison to PPP, phosphoketolase pathway can replenish acetyl-CoA availability for fatty acid metabolism and synthesis of lipids and membranes, which are important adaptations under hypoxia [62]. Thus, the phosphoketolase route may serve as another adaptation providing metabolic flexibility for wood-decaying fungi under alternating atmospheric and environmental conditions. Describing the role and function of the phosphoketolase pathway in filamentous fungi requires further investigation.

## Conclusions

Our results indicate that oxygen availability and substrate carbon source are both important regulators for expression of the secreted CAZy enzymes needed by wood-decaying fungi for degradation of lignocellulose and wood. Atmosphere appears to play a major role, especially in expression of cellulose-degrading CAZy genes, while the chemical composition of the lignocellulose substrate apparently influences expression of pectin-degrading CAZy genes. CAZy-regulating protein homologs differed between *Ascomycota* and *P. radiata*. This is not surprising, since the gene transcription factors in *Ascomycota* and *Basidiomycota* differ significantly [63] suggesting that the regulatory systems have evolved after these two fungal phyla diverged (about 570 million years ago) [7, 64]. Further studies are required to reveal how the enzyme cocktail produced by *P. radiata* in anaerobic or fermentative conditions could be utilized in industrial processes. *P. radiata* can utilize and ferment various carbohydrates. In addition, phosphoketolase pathway was induced under hypoxia. The role of this pathway requires further investigations to fully understand its role in eukaryotic organisms.

## Materials and methods

### Fungal isolate

*Phlebia radiata* 79 (FBCC 0043) was adopted as the target organism due to (i) its previous success in ethanol production from various lignocelluloses [15, 16]; (ii) the availability of a sequenced and fully annotated genome of high accuracy [19] (https://genome.jgi.doe.gov/Phlrad1/Phlrad1.home.html); (iii) the availability of analyzed transcriptomes and proteomes following wood decomposition under aerobic conditions [4]. A fungal isolate was deposited in the Microbial Domain Biological Resource Centre HAMBI FBCC sub-collection (https://kotka.luomus.fi/culture/fbcc) of the Helsinki Institute of Life Science, University of Helsinki, Finland. The isolate was cultivated and maintained on malt extract agar (2% w/v) at 25 °C in the dark throughout the study.

### Cultivation conditions

Solid-state cultivations were performed in 100-mL glass Erlenmeyer flasks that contained 4 g of dried spruce (*Picea abies*) wood as sawdust (particle size Ø < 2 mm) mixed with 1 g of dried and milled core board [15, 30]. The solid substrate mixture was dry autoclaved (121 °C, 15 min) prior to the addition of 20 mL of sterile 1% (w/v) yeast extract solution, pH 3. The yeast extract solution was prepared in water, and its acidity was adjusted with HCl prior to autoclaving (121 °C, 1 atm, 15 min). *P. radiata* was cultivated for 7 days on malt extract agar plates before a 6 mm fungal hyphae–agar plug was transferred into each flask (on top of the solid lignocelluloses). Cultivation flasks were sealed either with porous cellulose stoppers or with tight rubber plugs to create cultivation conditions with normal atmosphere or fermentative conditions, respectively. The atmosphere was analyzed by determining the proportions of O_2_, CO_2_, N_2_, CH_4_, C_2_H_4_ and N_2_O in the culture flask gas phase with Agilent 7890B gas chromatography equipped with thermal conductivity, flame ionization and electron capture detectors as previously reported [65]. The sample was obtained from the headspace of the cultivation vial with a syringe. The culture flasks were incubated without agitation at 25 °C in the dark, with three biological replicates. Samples were harvested from both atmospheric conditions on cultivation days 7, 14, and 21. Liquid medium cultivations with monosaccharides, polygalacturonic acid, or apple pectin as the carbon source were performed similarly under both atmospheric conditions. The medium contained 1% yeast extract pH 3 and 10 g/L of either glucose, xylose, mannose, galactose, polygalacturonic acid, or pectin. Samples were collected by separating the culture liquid from the solid substrate and mycelium by vacuum suction through a glass microfiber filter. Filtered culture liquid was adopted for enzyme activity assays and extracellular metabolite analyses by HPLC.

### RNA-Seq and transcriptome assembling

RNA was extracted and purified from mycelia growing in the solid lignocelluloses from three biological replicate cultures, under both atmospheric conditions, following previously described methods [4]. The yield of purified total RNA from “Spruce + board aerobic” cultivation was 183 µg (± 65) and 46 µg (± 6.4) from the cultivation “Spruce + board anaerobic”. The six RNA samples were sequenced at BGI (Hong Kong, People’s Republic of China). The quality trimmed RNA-Seq reads (number of reads varied between 22,944,957 and 23,415,252) of each sample were individually mapped against gene models of the genome assembly of *P. radiata* 79 [19] by STAR aligner version 2.4.1b as previously described [4].

### Transcriptome analyses

Aligned reads of each RNA-Seq set were counted using the HTSeq package of Chipster software version 3.15 guided by an annotation file containing genomic coordinates of the gene models [66, 67]. Differential gene expression between the transcriptomes was performed using DESeq 2 package [68] in the Chipster platform, as described previously [4]. Benjamini–Hochberg adjusted thresholds *p* < 0.05 and log2-fold change ≥ 1 or ≤ − 1 were used to determine significantly differentially expressed genes. Intracellular genes of core metabolism were analyzed by calculating fold change between cultivations ‘spruce + board anaerobic’ and ‘spruce + board aerobic’. Previously obtained transcriptomes of *P. radiata* on solid spruce wood (five RNA-Seq sets) [4] and on malt extract liquid medium (three RNA-Seq sets) [4] were included in the differential expression analysis of CAZy genes, in order to compare different substrates and atmospheric conditions, and to reveal regulative features. Mfuzz clustering of the gene expression data was performed using the Mfuzz 2.42.0 packages [21] in R version 3.5.2 (R Core Team 2019). Special attention was paid to annotated carbohydrate-active enzyme encoding genes (CAZy genes) of *P. radiata* [4, 19] along with the core metabolic genes annotated in this study. Hierarchical clustering and Venn diagrams were created using gplots 3.0.1.1 and Venn 1.7 packages, respectively, in R (R Core Team 2019). For hierarchical and Mfuzz clustering, the read counts were transformed into variance stabilized transformation (VST) values with Chipster software [66].

### Promoter region analysis

Promoter regions expanding 1000 bp upstream of the start codon on all annotated genes of *P. radiata* 79 genome assembly were downloaded from the JGI MycoCosm portal (genome site: https://genome.jgi.doe.gov/Phlrad1/Phlrad1.home.html). Enriched motifs within the promoter sequences were analyzed with the MEME Suite version 5.0.5 [69] package and MEME tool (meme-suite.org). The classic mode with default settings was used, apart from the occurrence being set to ‘any number of repetitions’, motif number set to 30, and motif size set from 5 to 20 nt. The occurrence of known motifs such as sequences for binding of specific transcription factors was scanned with the MEME Suite FIMO tool [70].

### Enzyme activity assays

Enzyme activity assays were performed in 96-well plate assay scale as previously described except for pectinase activity [71]. Pectinase activity was measured by using polygalacturonic acid as substrate and galacturonic acid as reference for product formation. Enzyme sample (20 µl) and 60 µl of substrate (0.5% polygalacturonic acid) were incubated for 10 min at 35 °C. The reaction was seized by adding 100 µl of dinitrosalicylic acid (DNS). The mixture was boiled for 5 min in a water bath, and the end product formation was measured at 540 nm with Spark multimode microplate reader (Tecan, Austria). Comparison of enzyme activities (mean values of three biological replicate values) was conducted with *t* test in the R-environment.

### Metabolic pathway prediction

We functionally annotated all genes involved in core metabolism to reveal, which metabolic pathways were affected by the differential expression of genes within each transcriptome. We did so by using BLASTP searches with homologs from well-studied fungi such as the budding yeast *S. cerevisiae*, Basidiomycota species *C. neoformans* and species of the genus *Aspergillus*. Functional annotation was targeted into genes involved in glycolysis, pentose phosphate pathway, pectin metabolism, ergosterol synthesis, fatty acid and triglyceride syntheses, fatty acid β-oxidation, and glyoxylate shunt. The predicted translated *P. radiata* proteins representing phosphoketolase and acetyl kinase were inspected in detail with their nearest homologs adopting ClustalW, and the protein models were 3D structurally studied by homology modeling using Phyre2 web portal for protein modeling, prediction and analysis [72]. An HPLC method was used for the quantitation of extracellular metabolites such as ethanol, methanol, acetate, glycerol, galacturonate, xylose, galactose, mannose and glucose as previously described [16]. Illustrations of the metabolic pathways were generated by Pathway tools 23.0 [73].

## Supplementary information

**Additional file 1: Table S1.** Atmosphere measurements of cultivation ‘Spruce + board Anaerobic’ and ‘Spruce + board aerobic’ at cultivation days 7, 14, 21 and 49. **Table S2.** Mfuzz center C-values of each cluster on each cultivation. Mfuzz classification of individual genes can be found in Additional file 1: Table S2 and Table S3. **Table S3.** Complete list of gene models, raw reads and DESeq 2-analysis of all 14 transcriptomes. Mfuzz cluster information of all 12,017 differentially expressed genes. **Table S4.** List and annotations of analysed 330 genes of primary metabolism and CAZy genes. Log2 fold change between ‘Spruce + board Anaerobic’ and ‘Spruce + board aerobic’—cultivations and promoter region analysis data of 11 different binding motif of common transcription factors. Mfuzz clusters and target/signal peptides of all studied core metabolism and CAZy genes. Annotation based on BlastP homology.

**Additional file 2.** Amino acid alignments of the translated protein candidates for phosphoketolase and acetyl kinase enzymes.

**Additional file 3.***P. radiata* phosphoketolase 3D structure. File can be viewed e.g. at https://bioinformatics.org/firstglance/fgij/index.htm.

**Additional file 4.** Overlay of phosphoketolase 3D protein structures. Green = *P. radiata* (plus.g11264), Gray =* Bifidobacterium breve* 3AHC.

## Data Availability

The RNA-Seq data presented in this publication is deposited in NCBI’s Gene Expression Omnibus and is accessible through GEO Series accession number GSE141153 (https://www.ncbi.nlm.nih.gov/geo/query/acc.cgi?acc=GSE141153). All other data generated or analyzed during this study are included in this published article and its additional information files.

## Abbreviations

CAZy: CAZyme carbohydrate-active enzyme
ROS: Reactive oxygen species
VST: Variance stabilizing transformation
LPMO: Lytic polysaccharide mono-oxygenase
LiP: Lignin peroxidase
MnP: Manganese peroxidase
SREBP: Sterol regulatory element binding protein
TCA-cycle: Tricarboxylic acid cycle
PEP: Phosphoenol pyruvate
DHAP: Dihydroxyacetone phosphate
PPP: Pentose phosphate pathway
