# Supplementary figures and images for: Hypoxia is regulating enzymatic wood decomposition and intracellular carbohydrate metabolism in filamentous white rot fungus
Source: Biotechnol Biofuels. 2020 Feb 24;13:26. doi: 10.1186/s13068-020-01677-0 (PMC7038570; doi:10.1186/s13068-020-01677-0)

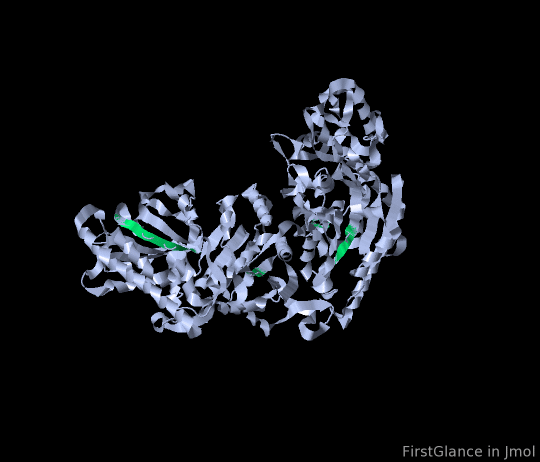

Supplement: Supplementary file 4 — Additional file 4. Overlay of phosphoketolase 3D protein structures. Green = P. radiata (plus.g11264), Gray = Bifidobacterium breve 3AHC. [file 13068_2020_1677_MOESM4_ESM.gif]
